# The Influence of Probiotic Supplementation on Depression, Anxiety, and Stress Level, as well as Inflammation, Anthropometric and Metabolic Parameters in Patients with Depressive Disorders - preliminary results of an RCT

**DOI:** 10.1192/j.eurpsy.2023.1782

**Published:** 2023-07-19

**Authors:** O. Gawlik-Kotelnicka, A. Skowrońska, A. Margulska, D. Strzelecki

**Affiliations:** 1 Department of Affective and Psychotic Disorders; 2 Department of Adolescent Psychiatry, Medical University of Lodz, Lodz, Poland

## Abstract

**Introduction:**

There is a huge need to search for new treatment options for depression but as well as its comorbidities. Particularly, depression and metabolic abnormalities often coexist, while a pathophysiological overlap, including microbiota changes, may play a role. Thus, the trials of microbiota interventions (e.g., probiotics) may establish a safe and easy-to-use treatment option as an adjunctive therapy in patients only partially responsive to pharmacological treatment.

**Objectives:**

The paper presents preliminary results of an RCT on the effect of probiotic supplementation on depression, anxiety and stress level, anthropometric, metabolic, and inflammatory parameters in adult patients with depressive disorders.

**Methods:**

The trial was a two-arm, parallel-group, prospective, randomized, double-blind, controlled design that included 43 participants and lasted 60 days. The probiotic preparation contained Lactobacillus helveticus Rosell®-52 and Bifidobacterium longum Rosell®-175 in the amount of 3 × 10^9^ colony forming units (CFU). We assessed depression level with Montgomery-Asberg Depression Rating Scale (MADRS), depressiveness, anxiety and stress level with 21-item version of Depression, Anxiety and Stress Scale (DASS-21), quality of life, blood pressure, body mass index and waist circumference, complete blood count, serum levels of C-reactive protein, high-density lipoprotein cholesterol, triglycerides, fasting glucose, selected secondary markers of inflammation and metabolic risk, as well as noninvasive biomarkers of liver fibrosis (APRI and FIB-4).

**Results:**

There were no differences in sociodemographic traits and psychometric questionnaires scores, as well as in anthropometric and basic laboratory findings between placebo and probiotic group at the start of the intervention period. Interestingly, there was a statistically significant improvement in MADRS score in both, placebo (p=0,010) and probiotic group (p=0,037) after intervention (see figure). The same finding was observed in total DASS-21 score as well as anxiety subscale of DASS-21. However, there were no differences in anthropometric, inflammation or metabolic laboratory parameters at the end of the study regardless of intervention.

**Image:**

**Figure:**
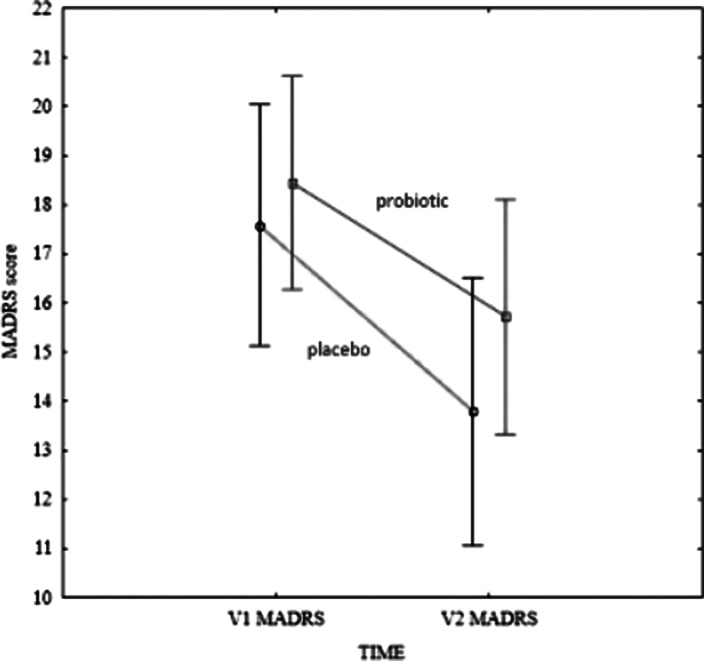

**Conclusions:**

Whilst probiotics may benefit some individuals who do not fully respond to antidepressant medications, our study did not show the superiority of probiotics over placebo in managing depressive and anxiety symptoms. However, the target clinical sample, as well as the intervention period and dosage of preparation for this intervention is not fully recognized. Moreover, larger clinical sample may be needed to detect differences between placebo and probiotics.

**Disclosure of Interest:**

None Declared

